# Supplementary material for: Heat adaptation of phage T7 under an extended genetic code
Source: Virus Evol. 2021 Dec 1;7(2):veab100. doi: 10.1093/ve/veab100 (PMC8923235; doi:10.1093/ve/veab100)
Supplement: veab100_Supp [file veab100_supp.zip › Sequencing_Analysis.zip!/Sequencing_Analysis/A3/evidence/JC_SIDE_2_59.html]

BRESEQ :: Evidence


| New junction evidence | | | | | | | | | | | |
| --- | --- | --- | --- | --- | --- | --- | --- | --- | --- | --- | --- |
|  | | seq id | position | reads (cov) | reads (cov) | score | skew | freq | annotation | gene | product |
| \* | ? | NC\_001604.1 | = 7530 | 2179 (1.250) | 29 (0.020) | 24/580 | 31.0 | 1.4% | intergenic (–/–) | *–/–* | –/– |
|  | ? | NC\_001604.1 | 8037 = | 2008 (1.190) |  |  |  |  | intergenic (–/–) | *–/–* | –/– |
| Rejected: Coverage evenness skew score above cutoff. | | | | | | | | | | | |
| Rejected: Frequency below/above cutoff threshold. | | | | | | | | | | | |

Only 100 of 2051 total aligned reads displayed.  

`TGCGTGTGGAGTATAGTTAACTGGTAATACGACTCACTAAAGGAGGTACACACCATGATGTACTTAATGCCATTACTCATCGTCATTGTAGGATGCCTTGCGCTCCACTGTAGCGATGATGATATGCCAGATGGTCACGCTTAATACGACTCACTAAAGGAGACACTATATGTTTCGACTTCATTACAACAAAAGCGTTAAGAATTTCACGGTTCGCCGTGCTGACCGTTCAATCGTATGTGCGAGCGAGCGCCGAGCTAAGATACCTCTTATTGGTAACACAGTTCCTTTGGCACCGAGCGTCCACATCATTATCACCCGTGGTGACTTTGAGAAAGCAATAGACAAGAAACGTCCGGTTCTTAGTGTGGCAGTGACCCGCTTCCCGTTCGTCCGTCTGTTACTCAAACGAATCAAGGAGGTGTTCTGATGGGACTGTTAGATGGTGAAGCCTGGGAAAAAGAAAACCCGCCAGTACAAGCAACTGGGTGTATAGCTTGCTTAGAGAAAGATGACCGTTATCCACACACCTGTAACAAAGGAGCTAACGATATGACCGAACGTGAACAAGAGATGATCATTAAGTTGATAGACAATAATG  >  NC_001604.1/7737‑8337`  

                                                                                                                                                                                                                                                                                                            `|`                                                                                                                                                                                                                                                                                                              
  `cGTGTGAAGTATAGTTAACTGGTAATACGACTCACTAAAGGAGGTACACACCATGATGTACTTAATGCCATTACTCATCGTCATTGTAGGATGCCTTGCGCTCCACTGTAGCGATGATGATATGCCAGATGGTAACGCTTAATACGACTCACTAAAGGAGACACTATATGTTTCGGCTTCATTACAACAAAAGCGTTAAGAATTTCACGGTTCGCCGTGCTGACCGTTCAATCGTATGTGCGAGCGAGCGCCGAGCTAAGATACCTCTTATTGGTAACACAGTTCCTTTGGCACCGAGCGt                                                                                                                                                                                                                                                                                                            >  1:135894/1‑301 (MQ=255)`  
       `ggtgTATAGTTAACTGGTAATACGAATCACTAAAAGAGGTACACACCATGATGTAATTAATGCCATTACTCATCGTCAGTGTAGGATGCCTTGCGCTCCACTGTAGCGATGATGATATGCCAGATGGTCACGCTTAATACGACTCACTAAATGAGACACTATATGTGTCGACTTCATTACAACAAAAGCGTTAAGAATTTCACTGTTCGCCGTGCTTACCGTTCAATCGTATGTACGAGCGAGCGCCGAGCTAAGATACCTCTTATTGGTAACACAGTTCCTTTGGCACCGAGCGTCcaca                                                                                                                                                                                                                                                                                                       <  1:334108/298‑1 (MQ=255)`  
        `gAGTATAGTTAACTGGTAATACGACTCACTAAAGGAGGTACACACCATGATGTACTTAATGCCATTACTCATCGTCATTGTAGGATGCCTTGCGCTCCACTGTAGCGATGATTCTATGCCAGATGGTCACGCTTAATACGACTCACTAAAGGAGACACTATATGTTTCGACTTCATTACAACAAAAGCGTTAAGAATTTCACGGTTCGCCGTGCTGACCGTTCAATCGTATGTGCGAGCGAGCGCCGAGCTAAGATACCTCTTATTGGTAACAAAGTTCCTTTGGCAACGAGCGTCCAcat                                                                                                                                                                                                                                                                                                      >  1:198628/1‑301 (MQ=255)`  
         `aatataGTTATCTGGTAATACGACTCACTAAAGGAGGTACACACCATGATGTACTTAATGCCATTACTCATCGTCATTGTAGGGAGCCTTGCGCTCCACTGTAGCGATGATGATATGCCAGGTGGGTACGCTGAATGCGACTCACTAAAGGAGACACTATATGTTTCGACTTCATTACAACAAAAGCGTTAAGAATTTCACGGTTCGCCGTGCTGACCGTTCAATCGTATGTGCGAGCGAGCGCCGAGCTAAGATACCTCTTATTGGTAACACAGTTCCTTTGGCACCGAGCGTCCAcatc                                                                                                                                                                                                                                                                                                     <  1:263877/299‑1 (MQ=255)`  
            `ataGTTAACTGGTAATACGACTCACTAAAGGAGGTACACACCATGATGTACTTAATGCCATTACTCATCGTCATTGTAGGATGCCTTGCGCTCCACTGTAGCGATGATGATATGCCAGATGGTCACGCTTAATACGACTCACTAAAGGAGACACTATATGTTTCGACTTCATTACAACAAAAGCGTTAAGAATTTCACGGTTCGCCGTGCTGACCGTTCAATCGTATGTGCGAGCGAGCGCCGAGCTAAGATACCTCTTATTGGTAACACAGTTCCTTTGGCACCGAGCGTccctgtctct                                                                                                                                                                                                                                                                                                  >  1:14017/1‑293 (MQ=255)`  
            `ataGTTAACTGGTAATACGACTCACTAAAGGAGGTACACACCATGATGTACTTAATGCCATTACTCATCGTCATTGTAGGATGCCTTGCGCTCCACTGTAGCGATGATGATATGCCAGATGGTCACGCTTAATACGACTCACTAAAGGAGACACTATATGTTTCGACTTCATTACAACAAAAGCGTTAAGAATTTCACGGTTCGCCGTGCTGACCGTTCAATCGTATGTGCGAGCGAGCGCCGAGCTAAGATACCTCTTATTGGTAACACAGTTCCTTTGGCACCGAGCGTCCACATCAtt                                                                                                                                                                                                                                                                                                  >  1:65871/1‑301 (MQ=255)`  
              `agagacagggTAATACGACTCACTAAAGGAGGTACACACCATGATGTACTTAATGCCATTACTCATCGTCATTGTAGGATGCCTTGCGCTCCACTGTAGCGATGATGATATGCCAGATGGTCACGCTTAATACGACTCACTAAAGGAGACACTATATGTTTCGACTTCATTACAACAAAAGCGTTAAGAATTTCACAGTTCGCCGTGCTGACCGTTCAATCGTATGTGCGAGCGAGCGCCGAGCTAAGATACCTCTTATTGGTAACACAGTTCCTTTGGCACCGAGCGTCCACATCATTAt                                                                                                                                                                                                                                                                                                <  1:215834/293‑1 (MQ=255)`  
              `aGTTAACTGGTAATACGACTCACGAAAGGAGGTACACACCATGATGTACTTAAGGCCATTACTCATCGTCATTGTAGGATGCCGTGCGCTCCACTATAGCGATGATGATATGCCAGATGGTCACGCTTAATACGACTAACTAAAGGAGACACTATATGTTTCGACTTCATTACAACAAAAGCGTTAAGAATTTCACGGTTCGCCGTGCTGACCGTTCAATCGTATGTGCGAGCGAGCGCCGAGCTAAGATACCTCTTATTGGTAACACAGTTCCTTTGGCACCGAGCGTCCACATCATTAt                                                                                                                                                                                                                                                                                                <  1:163500/301‑1 (MQ=255)`  
                  `ggctcggagatgtgtataagagacagggTACACACCATGATGTACTTAATGCCATTACTCATCGTCATTGTAGGATGCCTTGCGCTCCACTGTAGCGATGATGATATGCCAGATGGTCACGCTTAATACGACTCACTAAAGGAGACACTATATGTTTCGACTTCATTACAACAAAAGCGTTAAGAATTTCACGGTTCGCCATGCTGACCGTTCAATCGTATGTGCGAGCGAGCGCCGAGCTAAGATACCTCTTATTGGTAACACAGTTCCTTTGGCACCGAGCGTCCACATCATTATCAcc                                                                                                                                                                                                                                                                                            <  1:306543/275‑1 (MQ=255)`  
                  `aaCTGGTAATACGACTCACTAAAGGAGGTACACACCATGATGTACTTAATGCCATTACTCATCGTCATTGTAGGATGCCTTGCGCTCCACTGTAGCGATGATGATATGCCAGATGGTCACGCTTAATACGACTCACTAAAGGAGACACTATATGTTTCGACTTCATTACAACAAAAGCGTTAAGAATTTCACGGTTCGCCGTGCTGACCGTTCAATCGTATGTGCGAGCGAGCGCCGAGCTAAGATACCTCTTATTGGTAACACAGTTCCTTTGGCACCGAGCGTCCACATCATTATCAcc                                                                                                                                                                                                                                                                                            <  1:25713/301‑1 (MQ=255)`  
                    `cTGGTAATACGACTCACTAAAGGAGGTACACACCATGATGTACTTAATGCCATTACTCATCGTCATTGTAGGATGCCTTGCGCTCCACTGTAGCTATGATGATATGCCAGATGGTCACGCTTAATACGACTCACTAAAGGAGACACTATATGTTTCGACTTCATTACAACAAAAGCGTTAAGAATTTCACGGTTCGCCGTGCTGACCGTTCAATCGTATGTGCGAGCGAGCGCCGAGCTAAGATACCTCTTATTGGTAACACAGTTCCTTTGGCACCGAGCGTCCACATCATTATCACACg                                                                                                                                                                                                                                                                                          >  1:208961/1‑301 (MQ=255)`  
                       `ttAATACGACTAACTAAAGGAGGTACACACCATGATGTACTTAATGCCATTACTCATCGTCATTGTAGGATGCCTTGCGCTCCACTGTAGCGATGATGATATGCCAGATGGTCACGCTTAATACGACTCACTAAAGGAGACACTATATGTTTCGACTTCATTACAACAAAAGCGTTAAGAATTTCACGGTTCGCCGTGCTGACCGTTCAATCGTATGTGCGAGCGAGCGCCGAGCTAAGATACCTCTTATTGGTAACACAGTTCCTTTGGCACCGAGCGTCCACATCATTATCACCCgtgg                                                                                                                                                                                                                                                                                       <  1:329525/300‑1 (MQ=255)`  
                       `gTAATACGACTCACTAAAGGAGGTACACACCATGATGTACTTAATGCCATTACTCATCGTCATTGTAGGATGCCTTGCGCTCCACTGTAGCGATGATGATATGCCAGATGGTCACGCTTAATACGACTCACTAAAGGAGACACTATATGTTTCGACTTCATTACAACAAAAGCGTTAAGAATTTCACGGTTCGCCGTGCTGACCGTTCAATCGTATGTGCGAGCGAGCGCCGAGCTAAGATACCTCTTATTGGTAACACAGTTCCTTTGGCACCGAGCGTCCACATCATTATcctgtctct                                                                                                                                                                                                                                                                                       >  1:266442/1‑293 (MQ=255)`  
                         `aaTACGACTCACTAAAGGAGGTACACACCATGATGTACTTAATGCCATTACTCATCGTCATTGTAGGATGCCTTGCGCTCCACTGTAGCGATGATGATATGCCAGATGGTCACGCTTAATACGACTCACTAAAGGAGACACTATATGTTTCGACTTCATTACAACAAAAGCGTTAAGAATTTCACGGTTCGCCGTGCTGACCGTTCAATCGTATGTGCGAGCGAGCGCCGAGCTAAGATACCTCTTATTGGTAACACAGTTCCTTTGGCACCGAGCGTCCACATCATTATCACCCgtggtg                                                                                                                                                                                                                                                                                     <  1:91925/301‑1 (MQ=255)`  
                                  `cACTAAAGGAGGTACACACCATGATGTACTTAATGCCATTACTCATCGTCATTGTAGGATGCCTTGCGCTCCACTGTATCGATGATGATATGCCAGATGGTCACGCTTAATACGACTCACTAAAGGAGACACTATATGTTTCGACTTCATTACAACAAAAGCGTTAAGAATTTCACGGTTCGCCGTTCTGACCGTTCAATCGTATGTGCGAGCGCGCGCCGAGCTAAGATACCTCTTATTGGTAACACCATTCCTTTGTCACCGAGCGTCCACATCATTATCCCCCGTGGTGACTTTgaga                                                                                                                                                                                                                                                                            >  1:332379/1‑301 (MQ=255)`  
                                  `cACTAAAGGAGGTACACACCATGATGTACTTAATGCCATTACTCATCGTCATTGTAGGATGCCTTGCGCTCCACTGTAGCGATGATGATATGCCAGATGGTCACGCTTAATACGACTCACTAAAGGAGACACTATATGTTTCGACTTCATTACAACAAAAGCGTTAAGAATTTCACGGTTCGCCGTGCTGACCGTTCAATCGTATGTGCGAGCGAGCGCCGAGCTAAGATACCTCTTATTGGTAACACAGTTCCTTTGGCACCGAGCGTCCCCATCATTATctgtctcttatacacatctc                                                                                                                                                                                                                                                                            >  1:43384/1‑282 (MQ=255)`  
                                  `cACTAAAGGAGGTACACACCATGATGTACTTAATGCCATTACTCATCGTCATTGTAGGATGCCTTGCGCTCCACTGTAGCGATGATGATATGCCAGATGGTCACGCTTAATACGACTCACTAAAGGAGACACTATATGTTTCGACTTCATTACAACAAAAGCGTTAAGAATTTCACGGTTCGCCGTGCTGACCGTTCAATCGTATGTGCGAGCGAGCGCCGAGCTAAGATACCTCTTATTGGTAACACAGTTCCTTTGGCACCGAGCGTCCACATCATTATCACCCGTGGTGACTTTgaga                                                                                                                                                                                                                                                                            >  1:275834/1‑301 (MQ=255)`  
                                  `cACTAAAGGAGGTACACACCATGATGTACTTAATGCCATTACTCATCGTCATTGTAGGATGCCTTGCGCTCCACTGTAGCGATGATGATATGCCAGATGGTCACGCTTAATACGACTCACTAAAGGAGACACTATATGTTTCGACTTCATTACAACAAAAGCGTTAAGAATTTCACGGTTCGCCGTGCTGACCGTTCAATCGTATGTGCGAGCGAGCGCCGAGCTAAGATACCTCTTATTGGTAACACAGTTCATTTGGCACCGAGCGTCCACATCATTATCACCCGTGGTGACTTTgaga                                                                                                                                                                                                                                                                            >  1:109057/1‑301 (MQ=255)`  
                                  `cACTAAAGGAGGTACACACCATGATGTACTTAATGCCATTACTCATCGTCATTGTAGGATGCCTTGCGCTCCACTGTAGCGATGATGATATGCCAGATGATCACGCTTAATACGACTAACTAAAGGAGACACTATATGTTTCGAATTCATTACAACAAAAGCGTTAAGAATTTCACGGTTCGCCGTGCTGACCGTTCAATCGTATGTGCGAGCGAGCGCCGAGATAAGATACCTCTTATTGGTAACACAGTTCCTTTGGCACCGAGCGTCAACATCATTATCACCCGTGGTGAcctgactc                                                                                                                                                                                                                                                                            >  1:172687/1‑294 (MQ=255)`  
                                      `aaaGGAGGTACACACCATGATGTACTTAATGCCATTACTCATCGTCATTGAAGGATGCCTTGCGCTCCACTGTAGCGATGATGATATGCCAGATGGTCACGCTTAATACGACTCACTAAAGGAGACACTATATGTTTCGACTTCATTACAACAAAAGCGTTAAGAATTTCACGGTTCGCCATGCTGACCGTTCAATCGTATGTGCGAGCGAGCGCCGAGCTAAGATACCTCTTATTGGTAACACAGTTCCTTTGGCACCGAGCGTCAACATCATTATCACCCGTGGTGACTTTgagctgtc                                                                                                                                                                                                                                                                        >  1:142747/1‑296 (MQ=255)`  
                                            `ggTACACACCATGATGTACTTAATGCCATTACTCATCGTCATTGTAGGATGCCTTGCGCTCCACTGTAGCGATGATGATATGCCAGATGGTCACGCTTAATACGACTCACTAAAGGAGACACTATATGTTTCGACTTCATTACAACAAAAGCGTTAAGAATTTCACGGTTCGCCGTGCTGACCGTTCAATCGTATGTGCGAGCGAGCTCCGAGCTAAGATACCTCTTATTGGTAACACAGTTCCTTTGGCACCGAGCGTCCACATCATTATCACCCGTGGTGACTTTGAGAAAGCAATAGa                                                                                                                                                                                                                                                                  >  1:247324/1‑301 (MQ=255)`  
                                             `gTACACACCATGATGTACTTAATGCCATTACTCATCGTCATTGTAGGATGCCTTGCGCTCCACTGTAGCGATGATGATATGCCAGATGGTCACGCTTAATACGACTCACTAAATGAGACACTATATGTTTCGACTTCATTACAACAAAAGCGTTAAGAATTTCACGGTTCGCCGTGCTGACCGTTCAATCGTATGTGCGAGCGAGCGCCGAGCTAAGATACCTCTTATTGGTAACACAGTTCCTTTGGCACCGAGCGTCCACATCATTATCACCCGTGGTGACTTTGAGAAAGCAATAGAc                                                                                                                                                                                                                                                                 <  1:260523/301‑1 (MQ=255)`  
                                                   `acCATGATGTACTGAATGCCATTACTCATCGTCATTGTAGGATGCCTTGCGCTCCACTGTAGCGATGATGATAGGCCAGATGGTCACGCTTAATACGACTCACTAAAGGAGACACTATATGTTTCGACTTCATTACAACAAAAGCGTTAAGAATTTCACGGTTCGCCGTGCTGACCGTTCAATCGTATGTGCGAGCGATCGCCGAGCTAAGATACCTCTTATTGGTAACACAGTTCCTTTGGCACCGAGCGTCCACATCATTATCACCCGTGGTGACTTTGAGAAAGCAATAGACAAGaaa                                                                                                                                                                                                                                                           <  1:237857/301‑1 (MQ=255)`  
                                                      `atgatgTACTTAATGCCATTACTCATCGTCATTGTAGGATGCCTTGCGCTCCACTGTAGCGATGATGATATGCCAGATGGTCACGCTTAATACGACTCACTAAAGGAGACACTATATGTTTCGACTTCATTACAACAAAAGCGTTAAGAATTTCACGGTTCGCCGTGCTGACCGTTCAATCGTATGTGCGAGCGAGCGCCGAGCTAAGATACCTCTTATTGGTAACACAGTTCCTTTGGCACCGAGCGTACACATCATTATCACCCGTGGTGACTTTGGGAAAGCAATAGACAAGAACCGt                                                                                                                                                                                                                                                        >  1:309485/1‑301 (MQ=255)`  
                                                            `tACTTAATGCCATTACTCATCGTCATTGTAGGATGCCTTGCGTTCCACTTTAGCGATGAAGATATGCCATATGGTCACGCTTAATACTACTCACTAAAGGAGACACTATATGTTTCGACTTCATTACAACAAAAGCGTTAAGAATTTCACGGTTCGCCGTGCTGACCGTTCAATCGTATGTGCGAGCGAGCGCCGAGCTAAGATACCTCTTATTGGTAACACAGTTCCTTTGGCACCGAGCGTCCACATCATTATCACCCGTGGTGACTTTGAGAAAGCAATAGACAAGAAACGTCCGGtt                                                                                                                                                                                                                                                  <  1:27429/301‑1 (MQ=255)`  
                                                             `aCTTAATGCCATTACTCATCGTCATTGTAGGATGCCTTGCGCTCCACTGTAGCGATGATGATATGCCAGATGGTCACGCTTAATACGACTCACTAAAGGAGACACTATATGTTTCGACTTCATTACAACAAAAGCGTTAAGAATTTCACGGTTAGCCGTGCTGACCGTTCAATCGTATGTGCGAGCGAGCGCCGAGCTAAGATACCTCTTATTGGTAACACAGTTCCTTTGGCACCGAGCGTCCACATCATTATCACCCGTGGTGACTTTGAGAAAGCAATAGACAAGAAACGTCAGGTTc                                                                                                                                                                                                                                                 <  1:219069/301‑1 (MQ=255)`  
                                                              `cTTAATGCCATTACTCATCGTCATTGTAGGATGCCTTGCGCTCCACTGTAGCGATGATGATATGCCAGATGGTCACGCTTAATACGACTCACTAAAGGAGACACTATATGTTTCGACTTCATTACAACAAAAGCGTTAAGAATTTCACGGTTCGCCGTGCTGACCGTTCAATCGTATGTGCGAGCGAGCGCCGAGCTAAGATACCTCTTATTGGTAACACAGTTCCTTTGGCACCGAGCGTCCACATCATTATCACCCGTGGTGACTTTGAGAAAGCAATAGACAAGAAACGTCCGGTTCt                                                                                                                                                                                                                                                >  1:59241/1‑301 (MQ=255)`  
                                                                 `cccgCCGTTACTCATCGTCATTGGAGGATGCCTTGCGCTCCACTGTAGCGATGATGATATGCCAGATGGTCACGCTTAATACGACTCACTAAAGGAGACACTATATGTTTCGACTTCATTACAACAAAAGCGTTAAGAATTTCACGGTTCGCCGTGCTGACCGTTCAATCGTATGTGCGAGCGAGCGCCGAGCTAAGATACCTCTTATTGGTAACACAGTTCCTTTGGCACCGAGCGTCCACATCATTATCACCCGTGGTGACTTTGAGAAAGCAATAGACAAGAAACGTCCGGTTCTTAg                                                                                                                                                                                                                                             <  1:221638/298‑1 (MQ=255)`  
                                                                  `gtGCCATTACTCATCGTCATTGTAGGATGCCTTGCGCTCCACTGTAGCGATGATGATATGCCAGATGGTCACGCTTAATACGACTCACTAAAGGAGACACTATATGTTTCGACTTCATTACAACAAAAGCGTTAAGAATTTCACGGTTCGCCGTGCTGACCGTTCAATCGTATGTGCGAGAGAGCGCCGAGCTAAGATACCTCTTATTGGTAACACAGTTCCTTTGGCACCGAGCGTCCACATCATTATCACCCGTGGTGACTTTGAGAAAGCAATAGACAAGAAACGTCCGGTTCTTAgt                                                                                                                                                                                                                                            <  1:68306/300‑1 (MQ=255)`  
                                                                  `aagagacagcTCATCGTCATTGTAGGATGCCTTGCGCTCCACTGTAGCGATGATGATATGCCAGATGGTCACGCTTAATACGACTCACTAAAGGAGACACTATATGTTTCGACTTCATTACAACAAAAGCGTTAAGAATTTCACGGTTCGCCGTGCTGACCGTTCAATCGTATGTGCGAGCGAGCGCCGAGCTAAGATACCTCTTATTGGTAACACAGTTCCTTTGGCACCGAGCGTCCACATCATTATCACCCGTGGTGACTTTGAGAAAGCAATAGACAAGAAACGTCCGGTTCTTAgt                                                                                                                                                                                                                                            <  1:240048/292‑1 (MQ=255)`  
                                                                  `aTGCCATTACTCATAGTCATTGTAGGATGCCTGGCGCTCCACTGTAGCGATGATGATATGCCAGATGGTCACGCTTAATACGACTCACTAAAGGAGACACTATATGTTTCGACTTCATTACAACAAAAGCGTTAAGAATTTCACGGGTCGCCGTGCTGACCGTTCAATCGTATGTGCGAGCGAGCGCCGAGCTAAGATACCTCTTATTGGTAACACAGTTCCTTTGGCACCGAGCGTCCACATCATTATCACCAGTGGTGACTTTGAGAAAGCAATAGACAAGAAACGTCCGGTTCTTAgt                                                                                                                                                                                                                                            <  1:336300/301‑1 (MQ=255)`  
                                                                   `tGCCATTACTCATCGTCATTGTAGGATGCCTTTCGCTCCACTGTAGCGATGATGATATGCCAGATGGTCACGCTTAATACGACTCACTAAAGGAGACACTATATGTTTCGACTTCATTACAACAAAAGCGTTAAGAATTTCACGGTTCGCCGTGCTGACCGTTCAATCGTATGTGCGAGCGAGCGCCGAGCTAAGATACCTCTTATTGGTAACACAGTTCCTTTGGCACCGAGCGTCCACATCATTATCACCCGTGGTGACTTTGAGAAAGCAATAGACAAGAAACGTCCGGTTCTTAgtg                                                                                                                                                                                                                                           <  1:311915/301‑1 (MQ=255)`  
                                                                   `tGCCATTACTCATCGTCATTGTAGGATGCCTTGCGCTCCACTGTAGCGATGATGATATGCCAGATGGTCACGCTTAATACGACTCACTAAAGGAGACACTATATGTTTCGACTTCATTACAACAAAAGCGTTAAGAATTTCACGGTTCGCTGTGCTGACCGTTCAATCGTATGTGCGAGCGAGCGCCGAGCTAAGATACCTCTTATTGGTAACACAGTTCCTTTGGCACCGAGCGTCCACATCATTATCACCCGTGGTGACTTTGAGAAAGCAATAGACAAGAAACGTCCGGTTCTTAgtg                                                                                                                                                                                                                                           >  1:125553/1‑301 (MQ=255)`  
                                                                       `aTTACTCATCGTCATTGTAGGATGCCTTGCGCTCCACTGTAGCGATGATGATATGCCAGATGGTCACGCTTAATACGACTCACTAAAGGAGACACTATATGTTTCGACTTCATTACAACAAAAGCGTTAAGAATTTCACGGTTCGCCGTGCTGACCGTTCAATCGTATGTGCGAGCGAGCGCCGAGCTAAGATACCTCTTATTGGTAACACAGTTACTTTGGCACCGAGCGTCCACATCATTATCACCCGTGGTGACTTTGAGAAAGCAATAGACAAGAAACGTCCGGTTCTTAGTGTGGc                                                                                                                                                                                                                                       <  1:201933/301‑1 (MQ=255)`  
                                                                         `tACTCATCGTCATTGTAGGATGCCTTGCGCTCAACTGTAGCGATGATGATATGCCAGATGGTCACGCTTAATACGACTCACTAAAGGAGACACTTTATGTTTCGACTTCATTACAACAAAAGCGTTAAGAATTTCACGGTTCGCCGTGCTGACAGTTCAATCGTATGTGCGAGCGAGCACCGAGCTAAGATACCTCTTATTGGTAACACAGTTCCTTTGGCACCGAGCGTCCACATCATTATCACCCGTGGTGACTTTGAGAAAGCAATAGACAAGAAACGTCCGGTTCTTAGTGTGGCAg                                                                                                                                                                                                                                     <  1:282273/301‑1 (MQ=255)`  
                                                                                           `gATGCCTCGCGCTCCACTGTAGCGATGATGATATGCCAGATGGTCACGCTTAATACGACTCACTAAAGGAGACACTATATGTTTCGACTTCATTACAACAAAAGCGTTAAGAATTTCACGGTTCGCCGTGCTGACCGTTCAATCGTATGTGCGAGCGAGCGCCGAGCTAAGATACCTCTTATTGGTAACACAGTTCCTTTGGCACCGAGCGTCCACATCATTATCACCCGTGGTGACTTTGAGAAAGCAATAGACAAGAAACGTCCGGTTCTTAGTGTGGCAGTGACCCGCTTCCCGTTcg                                                                                                                                                                                                                   >  1:243325/1‑301 (MQ=255)`  
                                                                                             `tGCCTTGCGCTCCACTGTAGCGATGATGATATGCCAGATGGTCACGCTTAATACGACTCACTAAAGGAGACACTATATGTTTCGACTTCATTACAACAAAAGCGTTAAGAATTTCACGGTTCGCCGTGCTGACCGTTCAATCGTATGTGCGAGCGAGCGCCGAGCTAAGATACCTCTTATTGGTAACACAGTTCCTTTGGCACCGAGCGTCCACATCATTATCACCCGTGGTGACTTTGAGAAAGCAATAGACAAGAAACGTCCGGTTCTTAGTGTGGCAGTGACCCGCTTCCCGTTcgtc                                                                                                                                                                                                                 <  1:14597/301‑1 (MQ=255)`  
                                                                                               `aagagacagccACTGTAGCGATGATGATATGCCAGATGGTCACGCTTAATACGACTCACTAAAGGAGACACTATATGTTTCGACTTCATTACAACAAAAGCGTTAAGAATTTCACGGTTCGCCGTGCTGACCGTTCAATCGTATGTGCGAGCGAGCGCCGAGCTAAGATACCTCTTATTGGTAACACAGTTCCTTTGGCACCGAGCGTCCACATCATTATCACCCGTGGTGACTTTGAGAAAGCAATAGACAAGAAACGTCCGTTTCTTAGTGTGGCAGTGACCCGCTTCCCGTTcgtccg                                                                                                                                                                                                               <  1:53607/292‑1 (MQ=255)`  
                                                                                                      `cTCCACTGTAGCGATGATGATATGCCAGATGGTCACGCTTAATACGACTCACTAAAGGAGACACTATATGTTTCGACTTCATTACAACAAAAGCGTTAAGAATTTCACGGTTCGCCGTGCTGACCGTTCAATCGTATGTGCGAGCGAGCGCCGAGCTAAGATACCTCTTATTGGTAACACAGTTCCTTTGGCACCGAGCGTCCACATCATTATCACCCGTGGTGACTTTGAGAAAGCAATAGACAAGAAACGTCCGGTTCTTAGTGTGGCAGTGACCCGCTTCCCGTTctgtctcttatac                                                                                                                                                                                                        >  1:160489/1‑289 (MQ=255)`  
                                                                                                           `cTGTAGCGCTGATGATATGCCAGATGGTCACGCTTAATACGACTCACTAAAGGAGACACTATATGTTTCGACTTCATTACAACAAAAGCGTTAAGAATTTCACGGTTCGCCGTGCTGACCGTTCAATCGTATGTGCGAGCGAGCGCCGAGCTAAGATACCTCTTATTGGTAACACAGTTCCTTTGGCACCGAGCGTCCACATCATTATCACCCGTGGTGACTTTGAGAAAGCAATAGACAAGAAACGTCCGGTTCTTAGTGTGGCAGTGACCCGCTTCCCGTTCGTCCGTCTGTTACTCaa                                                                                                                                                                                                   >  1:298641/1‑301 (MQ=255)`  
                                                                                                           `cTGTAGCGATGATGATATGCCAGATGGTCACGCTTAATACGACTCACTAAAGGAGACACTATATGTTTCGACTTCATTACAACAAAAGCGTTAAGAATTTCACGGTTCGCCGTGCTGACCGTTCAATCGTATGTGCGAGCGAGCGCCGAGCTAAGATACCTCTTATTGGTAACACAGTTCCTTTGGCACCGAGCGTCCACATCATTATCACCCGTGGTGACTTTGAGAAAGCAATAGACAAGAAACGTCCGGTTCTTAGTGTGGCAGTGACCCGCTTCCCGTTCGTCCGTCTGTTACTCaa                                                                                                                                                                                                   >  1:151715/1‑301 (MQ=255)`  
                                                                                                                  `gatgatgatATGCCAGATGGTCACGCTTAATACGACTCACTAAAGGAGACACTATATGTTTCGACTTCATTACAACAAAAGCGTTAAGAATTTCACGGTTCGCCGTGCTGACCGTTCAATCGTATGTGCGAGCGAGCGACGAGCTAAGATACCTCTTATTGGTAACACAGTTCCTTTGGCACCGAGCGTCCACATCATTATCACCCGTGGTGACTTTGAGAAAGCAATAGACAAGAAACGTCAGGTTCTTAGTGTGGCAGTGACCCACTTCCCGTTCGTCCGTCTGTTACTCAAACGAATc                                                                                                                                                                                            >  1:166575/1‑301 (MQ=255)`  
                                                                                                                       `tgatATGCCAGATGGTCACGCTTAATACGACTCACTAAAGGAGACACTATATGTTTCGACTTCATTACAACAAAAGCGTTAAGAATTTCACGGTTCGCCGTGCTGACCGTTCAATCGTATGTGCGAGCGAGCGCCGAGCTAAGATACCTCTTATTGGTAACACAGTTCCTTTGGCACCGAGCGTCCACATCATTATCACCCGTGGTGACTTTGAGAAAGCAATAGACAAGAAACGTCCGGTTCTTAGTGTGGCAGTGACCCGCTTCCCGTTCGTCCGTATGTTACTCAAACGAATCAagga                                                                                                                                                                                       >  1:62083/1‑301 (MQ=255)`  
                                                                                                                       `tgatATGCCAGATGGTCACGCTTAATACGACTCACTAAAGGAGACACTATATGTTTCGACTTCATTACAACAAAAGCGTTAAGAATTTCACGGTTCGCCGTGCTGACAGTTCAATCGTATGTGCGAGCGAGCGCCGAGCTAAGATACCTCTTATTGGTAACACAGTTCCTTTGGCACCGAGCGTCCACATCATTATCACCCGTGGTGACTTTGAGAAAGCAATAGACAAGAAACGTCCGGTTCTTAGTGTGGCAGTGACCCGCTTCCCGTTCGTCCGTCTGTTACTCAAACGAATCAagga                                                                                                                                                                                       <  1:279155/301‑1 (MQ=255)`  
                                                                                                                         `gagatgtgtataagagacagtAATACGACTAACTAAAGGAGACACTATATGTTTCGACTTCATTACAACAAAAGCGTTAAGAATTTCACGGTTCGCCGTGCTGACCGTTCAATCGTATGTGCGAGCGAGAGCCGAGCTAAGATACCTCTTATTGGTAACACAGTTCCTTTGGCACCGAGCGTCCACATCATTATCACCAGTGGTGACTTTGAGAAAGCAATAGACAAGAAACGTCCGGTTCTTAGTGTGGCAGTGACCCGCTTCCCGTTCGTCCGTCTGTTACTCAAACGAATCAaggagg                                                                                                                                                                                     <  1:83595/281‑1 (MQ=255)`  
                                                                                                                              `ccAGATGGTCAAGCTTAATACGAATCACTAAAGGAGACCATATATTTTTCGACTTCATTACAACAAAAGCGTTAAGAATGTCACGGTTCGCCGTGCTGACCGTTCAATCGTATGTGCGAGCGAGCGCCGAGCTAAGATACCTCTTATTGGTAACACAGTTCCTTTGGCACCGAGCGTCCACATCATTATCACCCGTGGTGACTTTGAGAAAGCAATAGACAAGAAACGTCCGGTTCTTAGTGTGGCAGTGACACGCTTCCCGTTCGTCCGTCTGTTACTCAAACGAATCAAGGAGGTGTTc                                                                                                                                                                                <  1:71230/301‑1 (MQ=255)`  
                                                                                                                                `aGATGGTCACGCTTAATACGACTCACTAAAGGAGACACTATATGTTTCGACTTCATTACAACAAAAGCGTTAAGAATTTTACGGTTCGCCGTGCTGACCGTTCAATCGTATGTGCGAGCGAGCGCCGAGCTAAGATACCTCTTATTGGTAACACAGTTCCTTTGGCACCGAGCGTCCACATCATTATCACCCGTGGTGACTTTGAGAAAGCAATAGACAAGAAACGTCCGGTTCTTAGTGTGGCAGTGACCCGCTTCCCGTTCGTCCGACTGTTACTCAAACTAATCAAGGAGGTGTTcct                                                                                                                                                                              >  1:325997/1‑299 (MQ=255)`  
                                                                                                                                 `gATGGTCACGCTTAATACGACTCACTAAAGGAGAAACTATATGGTTCGACTTCATTACAACAAAAGCGTTAAGAATTTCACGGTTCGCCGTGCTGACCGTTCAATCGTATGTGCGAGCGAGCGCCGAGCTAAGATACCTCTTATTGGTAACACAGTTCCTTTGGCACCGAGCGTCCACATCATTATCACCCGTGGTGACTTTGAGAAAGCAATAGACAAGAAACGTCCGGTTCTTAGTGTGGCAGTGACCCGCTTCCCGTTCGTCCGTCTGTTACTCAAACGAATCAAGGAGGTGTTCTGa                                                                                                                                                                             <  1:27070/301‑1 (MQ=255)`  
                                                                                                                                       `cACGCTTAATACGACTCACTAAAGGAGACACTATATGTTTCTACTTCATTACAACAAAAGCGTTAAGAAGTTCACGGTTCGCCGTGCTGACCGTTCAATCGTATGTGCGAGCGAGCGCCGAGCTAAGATACCTCTTATTGGTAACACAGTTCCTTTGGCACCGAGCGTCCACATCATTATCACCCGTGGTGACTTTGAGAAAGCAATAGACAAGAAACGTCCGGTTCTTAGTGTGGCAGTGACCCGCTTCCCGTTCGTCCGTCTGTTACTCAAACGAATCAAGGAGGTGTTCTGATGGGAc                                                                                                                                                                       <  1:73705/301‑1 (MQ=255)`  
                                                                                                                                        `aaagagacagcGACTCACTAAAGGAGACACTATATGTTTCGACTTCATTACAACAAAAGCGTTAAGAATTTCACGGTTCGCCGTGCTGACCGTTCAATCGTATGTGCGAGCGAGCGCCGAGCTAAGATACCTCTTATTGGTAACACAGTTCCTTTGGCACCGAGCGTCCACATCATTATCACCCGTGGTGACTTTGAGAAAGCAATAGACAAGAAACGTCCGGTTCTTAGTGTGGCAGTGACCCGCTTCCCGTTCGTCCGTCTGTTACTCAAACGAATCAAGGAGGTGTTCTGATGGGACt                                                                                                                                                                      <  1:185456/291‑1 (MQ=255)`  
                                                                                                                                         `cGCTTAATACGACTCACTAAAGGAGACACTATATGTTTCGACTTCATTACAACAAAAGCGTTAAGAATTTCACGGTTCGCCGTGCTGACCGTTCAATCGTATGTGCGAGCGAGCGCCGAGCTAAGATACCTCTTATTGGTAACACAGTTCCTTTGGCACCGAGCGTCCACATCATTATCACCCGTGGTGACTTTGAGAAAGCAATAGACAAGAAACGTCCGGTTCTTAGTGTGGCAGTGACCCGCTTCCCGTTCGTCCGTCTGTTACTCAAACGAATCAAGGAGGTGTTCTGATGGGAcct                                                                                                                                                                     >  1:46683/1‑299 (MQ=255)`  
                                                                                                                                           `cTTAATACGACTCACTAAAGGAGACACTATATGTTTCGACTTCATTACAACAAAAGCGTTAAGAATTTCACGGTTCGCCGTGCTGACCGTTCAATCGTATGTGCGAGCGAGCGCCGAGCTAAGATACCTCTTATTGGTAACACAGTTCCTTTGGCACCGAGCGTCCACATCATTATCACCCGTGGTGACTTTGAGAAAGCAATAGACAAGAAACGTCCGGTTCTTAGTGTGGCAGTGACCCGCTTCCCGTTCGTCCGTCTGTTACTCAAACGAATCAAGGAGGTGTTCTGATGGGACTGtt                                                                                                                                                                   >  1:115701/1‑301 (MQ=255)`  
                                                                                                                                                 `aCGACTCACTAAAGGAGACACTATATGTTACGACTTCATTACAACAAAAGAGTTAAGAGTTTCACGGTTCGCCGTGCTGACCGTTCAAGCATATGTGCGAGCGAGCGCCGAGCTAAGATACCTCTTAGTGGTAACACAGTTCCTTTGGCACCGAGCGTCCACATCATTATCACCCGTGGTGACTTTGAGAAAGCAATAGACAAGAAACGTCCGGTTCTTAGTGTGGCAGTGACCCGCTTCCCGTTCGTCCGTCTGTTACTCAAACGAATCAAGGAGGTGTTCTGATGGGACTGTTAGATgg                                                                                                                                                             <  1:204825/301‑1 (MQ=255)`  
                                                                                                                                                  `cGACTCACTAAAGGAGACACTATATGTTTCGACTTCATTACAACAAAAGCGTTAAGAATTTCACGGTTCGCCGTGCTAACCGTTCAATCGTATGTGCGAGCGAGCGCCGAGCTAAGATACCTCTTATTGGTAACACAGTTCCTTTGGCACCGAGCGTCCACATCATTATCACCCGTGGTGACTTTGAGAAAGCAATAGACAAGAAACGTCCGGTTCTTAGTGTGGCAGTGACCCGCTTCCCGTTCGTCCGTCTGTTACTCAAACGAATCAAGGAGGTGTTCTGATGGGACTGTTAGATGGt                                                                                                                                                            <  1:2502/301‑1 (MQ=255)`  
                                                                                                                                                      `gcACGAAAGGAGACACTGTATGTTGCGTCATCATGACAAGAAAAGCGTTAAGGATTTCACGGTTCGTCGTTCTGACCATTAAATCGTAAGTGCGAGCGAGAGCCGAGCGAAGATACCTCTTATTGGTAACACAGTTCCTTTGGCACCGAGCGTCCACATCATTATCACCCGTGGTGACTTGAAGAAAGCAATAGACAAGAAACGTCCGGTACTTAGTGTGGCAGTGACCCGCTTCCCGTTCGTCCGTCTGTGACTCAAACGAATCAAGGAGGTGTTCTGATGGGACTGTTAGATGGTGAAg                                                                                                                                                        <  1:295109/300‑1 (MQ=255)`  
                                                                                                                                                       `cACTAAAGGAGACACTATATGTTTCGACTTCATTACAACAAAAGCGTTAAGAATTTCACGGTTCGCCGTGCTGACCGTTCAATCGTATGTGCGAGCGAGCGCAGAGCTAAGATACCTCTTATTGGTAACACAGTTCCTTTGGCACCGAGCGTCCACATCATTATCACCCGTGGTGACTTTGAGAAAGCAATAGACAAGAAACGTCCGGTTCTTAGTGTGGCAGTGACCCGCTTCCCGTTCGTCCGTCTGTTACTCAAACGAATCAAGGAGGTGTTCTGATGGGACTGTTAGAtctgtctct                                                                                                                                                       >  1:175169/1‑293 (MQ=255)`  
                                                                                                                                                        `gtataagaaacagcTATATGTTTCGACTTCATTACAACAAAAGCGTTAAGAATTTCACGGTTCTCCGTGCTGACCGTTCAATCGTATGTGCGAGCGAGCGCCGAGCTAAGATACCTCTTATTGGTAACACAGTTACTTTGGCACCGAGCGTCCACATCATTATCACCCGTGGTGACTTTGAGAAAGCAATAGACAAGAAACGTCCGGTTCTTAGTGTGGCAGTGACCCGCTTCCCGTTCGTCCGTCTGTTACTCAAACGAATCAAGGAGGTGTTCTGATGGGACTGTTAGATGGTGAAGcc                                                                                                                                                      <  1:338961/288‑1 (MQ=255)`  
                                                                                                                                                           `aacggAGACACTATATGTTTCGACTTCATTACAACAAAAGCGTTAAGAATTTCACGGTTCGCCGTGCTGACCGTTCAATCGTATGTGCGAGCGAGCGCCTACCTAAGATACCTCTTATTGGTAACACAGTTCCTTTGGCACCGCGCGCCCACATCATTATCACCCGTGGTGACTTTGAGAAAGCACTAGACAAGAAACGTCCGGTTCTTAGTGTGGCAGTGACCCGCTTCCAGTTCGTCCGTCTGTTACTCAACCGAATCAAGTAGCTGTTCTGATGGGACTGTTAGATGGTGAAGCCTgg                                                                                                                                                   >  1:290835/4‑301 (MQ=255)`  
                                                                                                                                                                `agaCACTATATGTTTCGACTTCATTACAACAAAAGCGTTAAGAATTTCACGGTTCGCCGTGCTGACCGTTCAATCGTATGTGCGAGCGAGCGCCGAGCTAAGATACCTCTTATTGGTAACACAGTTCCTTTGGCACCGAGCGTCCACATCATTATCACCCGTGGTGACTTTGAGAAAGCAATAGACAAGAAACGTCCGGTTCTTAGTGTGGCAGTGACCCGCTTCCCGTTCGTCCGTCTGTTACTCAAACGAATCAAGGAGGTGTTCTGATGGGACTGTTAGATGGTGAAGCCTGGGaaaa                                                                                                                                              <  1:212877/301‑1 (MQ=255)`  
                                                                                                                                                                  `acacTATATGTTTCGACTTCATTACAACAAAAGCGTTAAGAATTTCACGGTTCGCCGTGCTGACCGTTCAATCGTATGTGCGAGCGAGCGCCGAGCTAAGATACCTCTTATTGGTAACACAGTTCCTTTGGCACCGAGCGTCCACATCATTATCACCCGTGGTGACTTTGAGAAAGCAATAGACAAGAAACGTCCGGTTCTTAGTGTGGCAGTGACACGCTTCCCGTTCGTCCGTCTGTTACTCAAACGAATCAAGGAGGTGTTCTGATGGGACTGTTAGATGGTGAAGCCTGGGAAAAAg                                                                                                                                            <  1:195621/301‑1 (MQ=255)`  
                                                                                                                                                                   `cacTATATGTTTCGACTTCATTACAACAAAAGCGTTAAGAATTTCACGGTTCGCCGTGCTGACCGTTCAATCGTATGTGCGAGCGAGCGCCGATCTAAGATACCTCTTATTGGTAACACAGTTCCTTTGGCACCGAGCGTCCACATCATTATCACCCGTGGTGACTTTGAGAAAGCAATAGACAAGAAACGTCCGGTTCTTAGTGTGGCAGTGACCCGCTTCCCGTTCGTCCGTCTGTTACTCCAACGAATCCAGGAGGTGTTCTGATGGGACTCGTAGATGGTGAATCCTGGGACAAAGa                                                                                                                                           >  1:37611/1‑301 (MQ=255)`  
                                                                                                                                                                   `cacTATATGTTTCGACTTCATTACAACAAAAGCGTTAAGAATTTCACGGTTCGCCGTGCTGACCGTTCAATCGTATGTGCGAGCGAGCGCCGAGCTAAGATACCTCTTATTGGTAACACAGTTCCTTTGGCACCGAGCGTCCACATCATTATCACCCGTGGTGACTTTGAGAAAGCAATAGACAAGAAACGTCCGGTTCTTAGTGTGGCCGTGACCCGCTTCCCGTTCGTCCGTCTGTTACTCAAACGAATCAAGGAGGTGTTCTGATTGGACTGTTCGATGGTGAAGCCTGGGAAAAAGa                                                                                                                                           >  1:187816/1‑301 (MQ=255)`  
                                                                                                                                                                   `agacagatGTTTCGACTTCATTACAACAAAAGCGTTAAGAATTTCACGGTTCGCCGTGCTGACCGTTCAATCGTATGTGCGAGCGAGCGCCGAGCTAAGATACCTCTTATTGGTAACACAGTTCCTTTGGCACCGAGCGTCCACATCATTATCACCCGTGGTGACTTTGAGAAAGCAATAGACAAGAAACGTCCGGTTCTTAGTGTGGCAGTGACCCGCTTCCCGTTCGTCCGTCTGTTACTCAAACGAATCAAGGAGGTGTTCTGATGGGACTGTTAGATGGTGAAGCCTGGGAAAAAGa                                                                                                                                           <  1:40496/295‑1 (MQ=255)`  
                                                                                                                                                                   `aacTAGATGTTTCGACTTCATTACAACAAAAGCGTTAAGAATTTCACGGTTCGCCGTGCTGACCGTTCAATCGTATGTGCGAGCGAGCGCCGAGCTAAGATACCTCTTATTGGTAACACAGTTCCTTTGGCACCGAGCGTCCACATCATTATCACCCGTGGTGACTTTGAGAAAGCAATAGACAAGAAACGTCCGGTTCTTAGTGTGGCAGTGACCCGCTTCCCGTTCGTCCGTCTGTTACTCAAACGAATCAAGGAGGTGTTCTGATGGGACTGTTAGATGGTGAAGCCTGGGAAAAAGa                                                                                                                                           <  1:193297/300‑1 (MQ=255)`  
                                                                                                                                                                     `cTATATGTTTCGACTTCCTTACAACAAAAGCGTTAAGAATTTCACGGTTCGCCGTGCTGACCGTTCAATCGTATGTGCGAGCGATCGCCGAGCTAAGATACCTCTTATTGGTAACACAGTTCCTTTGGCACCGAGCGTCCACATCATTATCACCCGTGGTGACTTTGAGAAAGAAATAGACACGCACCGTCCGGTTCTTAGTGTGGCTGTGACCCGCTTCCAGTTCGTCCGTCTGTTACTCAAACGAATCAAGGAGGTGTTCTGATGGGACTGTTAGATGGTGAAGCATggtcaagtgaaa                                                                                                                                         >  1:106414/1‑291 (MQ=255)`  
                                                                                                                                                                           `gTTTCGACTTCATTACAACAAAAGCGTTAAGAATTTCACGGTTCGCCGTGCTGACCGTTCAATCGTATGTGCGAGCGAGCGCCGAGCTAAGATACCTCTTATTGGTAACACAGTTCCTTTGGCACCGAGCGTCCACATCATTATCCCCCGTGGTGACTTTTAGAAAGCAATAACCAAGAAACGTCCGGTTCTTAGTGTGGCAGTGACCCGCTTCCCGTTCGTCCGTCTGTTCCTCAAACGAATCAAGGAGGTGTTCTGATGGGACTTTTTGATGGTGAATCTTGGGAAAAAGAAAACCCGc                                                                                                                                   >  1:128685/1‑301 (MQ=255)`  
                                                                                                                                                                           `gTTTCGACTTCATTACAACAAAAGCGTTAAGAATTTCACGGTTCGCCGTGCTGACCGTTCAATCGTATGTGCGAGCGAGCGCCGAGCTAAGATACCTCTTATTGGTAACACAGTTCCTTTGGCACCGAGCGTCCACATCATTATCACCCGTGGTGACTTTGAGAAAGCAATAGACAAGAAACGTCCGGTTCTTAGTGTGGCAGTGACCCGCTTCCCGTTCGTCCGTCTGTTACTCAAACGAATCAAGGAGGTGTTCTGATGGGACTGTTAGATGGTGAAGCCTGGGAAAAAGAAAACCCGc                                                                                                                                   >  1:34466/1‑301 (MQ=255)`  
                                                                                                                                                                                          `caacaaAAGCGTTAAGAATTTCACGGTTCGCCGTGCTGACCGTTCAATCGTATGTGCGAGCGAGCGCCGAGCTAAGATACCTCTTATTGGTAACACAGTTCCTTTGGCACCGAGCGTCCACATCATTATCACCCGTGGTGACTTTGAGAAAGCAATAGACAAGAAACGTCCGGTTCTTAGTGTGGCAGTGACCAGCTTCCCGTTCGTCCGTCTGTTACTCAAACGAATCAAGGAGGTGTTCTGATGGGACTGTTAGATGGTGAAGCCTGGGAAAAAGAAAACCCGCCAGTACAAGCAACTg                                                                                                                    >  1:85519/1‑301 (MQ=255)`  
                                                                                                                                                                                            `acaaAAGCGTTAAAAATTTCACGGTTCGCCGTGCTGACCGTTCAATCGTATGTGCGAGCGGCCGCCGAGCTAAGATACCTCTTATTGGTAACACAGTTCCTTTGGCACCGAGCGTCCACATCATTATCACCCGTGGTGACTTTGAGAAAGCAATAGACAAGAAACGTCCGGTTCTTAGTGTGGCAGTGACCCGCTTCCCGTTCGTCCGTCTGTTACTCAAACGAATCAAGGAGGTGTTCTGATGGGACTGTTAGATGGTGAAGCCTGGGAAAAAGAAAACCCGCCAGTACAAGCAACTggg                                                                                                                  <  1:177857/301‑1 (MQ=255)`  
                                                                                                                                                                                                 `aGCGTTAAGAAGTGCACGGGTCGCCGTGCTGACCGTTCAATCGTATGGGCGAGCGAGCGCCGAGCTAAGACACCTCTTATTGGTAACACAGTTCCTTTGGCACCGAGCGGCCACATCATTATCACCCGTGGTGACTTGAAGAAAGCAATAGACAAGAAACGTCCGGTTCTTAGGGTGGCAGTGACCCGCTTCCCGTTCGTCCGTCTGTTACTCAAGCGAATCAAGGAGGTGTTCTGATGGGACTGTTAGATGGTGAAGCCTGGGAAAAAGAAAACCCGCCAGTACAAGCAACTGGGTGtat                                                                                                             <  1:119665/301‑1 (MQ=255)`  
                                                                                                                                                                                                   `cGTTATGAATTTCACGGTTAGCCGTGATTACCGTTCAAGCGTATGTGGGAGCGAGCGCCGGGCTAGGATACCTCTGATTGGTAACACAGTACCTTTGGCACGGAGCGTCCACATCATTATCACCCGGGGTGACTTTGAGAAAGCAATAGACAAGAAACGTCCGGTGCTGATGGTGGCAGTGACCCGCGTCCCGTTCGTCCGTCTGTTACTCAAACGAATCAAGGAGGTGTTCTGATTGGACTGTTAGATGGTGAAGCCTGGGAAAAAGAAACCCCGCCAGTACAAGCAACTGGGTGTATAg                                                                                                           <  1:190136/301‑1 (MQ=255)`  
                                                                                                                                                                                                    `attAAGAATTTCACGGTTCGCCGTGCTGACCGTTCAATCGTATGGGAGAGCGAGCGCCGAACTAAGATGCCTCTTAATGGTAACACGGTTCCTTGGGAACCGAGCGACCACATCATTAACACCCGTAGTGACTTTGAGAAAGCAATGGACAAAAAACGTAGGGTTCTTAGTGTGGCAGTGGCCCGCTTCCCGTTCCTCCGTCTGTTACACAAACGAATCAAGGAGGTGTTCTGATGGGACTGTTAGATGGTGAAGCCTGGAAAAAAGAAAACCCGCCAGTACAAGCAACTGGGTGTATAgc                                                                                                          <  1:25295/300‑1 (MQ=255)`  
                                                                                                                                                                                                         `gAATTTCACGGTTCGCCGTGCTGACCGTTCAATCGTATGTGCGAGCGAGCGCCGAGCTAAGATACCTCTTATTGGTAACACAGTTCCTTTGGCACCGAGCGTCCACATCATTATCACCCGTGGTGACTTTGAGAAAGCAATAGACAAGAAACGTCCGGTTCTTAGTGTGGCAGTGACCCGCTTCACGTTCGTCCGTCTGTTACTCAAACGAATCAAGGAGGTGTTCTGATGGGACTGTTAGATGGTGAAGCCTGGGAAAAAGAAAACTCGCCAGTACAAGCAACTGGGTGTATAgcttgct                                                                                                     >  1:169498/1‑301 (MQ=255)`  
                                                                                                                                                                                                            `tttCACGGTTCGCCGTGCTGACCGTTCAATCGTATGTGCGAGCGAGCGCCGAGCTAAGATACCTCTTATTGGTAACACAGTTCCTTTGGCACCGAGCGTCCACATCATTATCACCCGTTGTGACTTTGAGAAAGCAATAGACAAGAAACGTCCGGTTCTTAGTGTGGCAGTGACCCGCTTCCCGTTCGTCCGTCTGTTACTCAAACGAATCAAGGAGGTGTTCTGATGGGACTGTTAGATGGTGAAGCCTGGGAAAAAGAAAACCCGCCAGTACAAGCAACTGGGTGTATAGCTTGCTTag                                                                                                  <  1:95662/301‑1 (MQ=255)`  
                                                                                                                                                                                                               `agcGGTTCGCCGTGCTGACCGTTCAATCGTATGTGCGAGCGAGCGCCGAGCTAAGATACCTCTTATTGGTAACAAAGTTCATTTGGCACCGAGCGTCCACATCATTATCACCCGTGGTGACGTTGAGAAAGCAATAGACAAGAAACGTCCGGTTCTTAGTGTGGCAGTGACCCGCTTCCCGTTCGTCCGTCTGTTACTCAAACGAATCAAGGAGGTGTTCTGATGGGACTGTTAGATGGTGAAGCCTGGGAAAAAGAAAACCCGCCAGTACAAGCAACTGGGTGTATAGCTTGCTTAgaga                                                                                               <  1:103386/299‑1 (MQ=255)`  
                                                                                                                                                                                                                 `cGGTTCGCCGTGCTGACCGTTCAATCGTATGTGCGAGCGAGCGCCTAGCTAAGATACCTCTTATTGGTAACACAGTTCCTTTGGCACCGAGCGTCCACATCATTATCACCCGTGGTGACTTTTAGAAAGCAATAGACAAGAAACGTCCGGTTCTTAGTGTGGCAGTGACCCGCTTCCCGTTCGTCCGTCTGTTACTCAAACGAATCAAGGAGGTGTTCTGATGGGACTGTTAGATGGTGAAGCCTGGGAAAAAGAAAATCCGCCAGTACAAGCAACTGGGTGTATAGCTTGCTTAGAGaaa                                                                                             >  1:157420/1‑301 (MQ=255)`  
                                                                                                                                                                                                                   `gTTCGCCGTGCTTACCGTTCATTCGTATGTGCGAGCGAGCGCCGAGCTAAGATACCTCTTATTGGTAACACAGTTCCTTTGGCACCGAGCGTCCACATCATTATCACCCGTGGTGACTTTGAGAAAGCAATTGACAAGAACCGTCCGGTTCTTAGTGTGGCAGTGACACGCTTCCCGTTCGTCCGTCTGTTACTCAAACGAATCAAGGAGGTGTTCTGATTGGACTGTTAGATGGTGAAGCCTGGGAAAAAGAAAACCCGCCAGTACAAGCAACTGGGTGTATAGCTTGCTTAGAGAAAGa                                                                                           <  1:322973/301‑1 (MQ=255)`  
                                                                                                                                                                                                                    `ttCGCCTTCCGGACCTTTCAGTCTGATGTGCAGGCGAGCGCCGAGCTAAGATACCTTTTATTGGTAACACATTTCCTTTGGCACCGAGCGTCCACATCATTATCACCCGTGGTAACTTTGAGAAAGCAATAGACAAGAAACGTCCGGTTCTTAGTGTGGCAGTGACCCGATTCCCGTTCGTCCGTCTGTTACTCAAACGAATCAAGGAGGTGTTCTGATGGGACTGTTAGATGGTGAAGCCTGGGAAAAAGAAAACCCGCCAGTACAAGCAACTGGGTGTATAGCTTGCTTAGAGAAAGAt                                                                                          <  1:284977/301‑1 (MQ=255)`  
                                                                                                                                                                                                                               `gACCGTGCAATCGTATGTGCGAGCGAGCGCCGAGCTAAGATACCTCTTATTGGTAACACAGTTCCTTTGGCACCGAGCGTCCACATCATTATCACCCGTTGTGACTTTGAGAAAACAATATACATGAAACGTCCGGTTCTTAGTGTGGCAGTGACCCGCTTCCCGTTCGTCCGTCTGTTACTCAAACGAATCAAGGAGGTGTTCTGATGGGACTGTTAGATGGTGAAGCCTGGGAAAAAGAAAACCCGCCAGTACAAGCAACTGGGTGTATAGCTTGCTTAGAGAAAGATGACCGTTATcc                                                                               <  1:155256/301‑1 (MQ=255)`  
                                                                                                                                                                                                                                        `aTCGTATGTGCGAGCGAGCGCCGAGCTAAGATACCTCTTATTGGTAACACAGTTCCTTTGGCACCGAGCGTCCACATCATTATCACCCGTGGTGACTTTGAGAAAGCAATAGACAAGAAACGTCCGGTTCTTCGTGTGGCAGTGACCCGCTTCCCGTTCGTCCGTCTGTTACTCAAACGAATAAAGGAGGTGTTCTGATGGGACTGTTAGATGGTGAAGCCTGGGAAAAAGAAAACCCGCCAGTACAAGCAACTGGGTGTATAGCTTGCTTAGAGAAAGATGACCGTTATCCACACACCtt                                                                      >  1:181854/1‑300 (MQ=255)`  
                                                                                                                                                                                                                                         `tCGTATGTGCGAGCGGGCGCCGAGCTAAGATACCTCTTATTGGTAACACAGTTCCTTTGGCACCGAGCGTCCACATCATTATCACCCGTGGTGACTTTGAGAAAGCAATAGACAAGAAACGTCCGGTTCTTAGTGTGGCAGTGACCCGCTTCCCGTTCGTCCGTCTGTTACTCAAACGAATCAAGGAGGTGTTCTGATGGGACTGTTAGATGGTGAAGCCTGGGAAAAAGAAAACCCGCCAGTACAAGCAACTGGGTGTATAGCTTGCTTAGAGAAAGATTACCGTTATCCACACACCTGt                                                                     <  1:122140/301‑1 (MQ=255)`  
                                                                                                                                                                                                                                         `tCGTATGTGCGAGCGAGCGCCGAGCTAAGATACCTCTTATTTGTAACACAGTTCCTTTGGCACCGAGCGTCCACATCATTATCACCCGTGGTGACTTTGAGAAAGCAATAGACAAGAAACGTCTGGTTCTTAGTGTGGCAGTGACCCGCTTCCCGTTCGTCCGTCTGTTACTCAAACGAATCAAGGAGGTGTTCTGATGGGACTGTTAGATGGTGAAGCCTGGGAAAAAGAAAACCCGCCAGTACAAGCAACTGGGTGTATAGCTTGCTTAGAGAAAGATGACCGTTATCCACACACCTGt                                                                     <  1:112320/301‑1 (MQ=255)`  
                                                                                                                                                                                                                                             `aTGTGCGAGCGAGCGCCGAGCTAAGATACCTCTTATTGGTAACACAGTTCCTTTGGCACCGAGCGTCCACATCATTATCACCCGTGGTGACTTTGAGAAAGCAATAGACAAGAAACGTCCGGTTCTTAGTGTGGCAGTGACCCGCTTCCCGTTCGTCCGTCTGTTACTCAAACGAATCAAGGAGGTGTTCTGATGGGACTGTTAGATGGTGAAGCCTGGGAAAAAGAAAACCCGCCAGTACAAGCAACTGGGTGTATAGCTTGCTTAGAGAAAGATGACCGTTATCCACACACCTGTAACa                                                                 <  1:148039/301‑1 (MQ=255)`  
                                                                                                                                                                                                                                                 `gatgtgtataagggacagaaGATACCTCTTATTGGTAACAAAGTTCCTTTGGCACCGAGCGTCCACATCATTATCACCCGTGGTGACTTTGAGAAAGCAATAGACAAGAAACGTCCGGTTCTTAGTGTGGCAGTGACCCGCTTCCCGTTCGTCCGTCTGTTACTCAAACGAATCAAGGAGGTGTTCTGATGGGACTGTTAGATGGTGAAGCCTGGGAAAAAGAAAACCCGCCAGTACAAGCAACTGGGTGTATAGCTTGCTTAGAGAAAGATGACCGTTATCCACACACCTGTAACAAAgg                                                             <  1:234821/283‑1 (MQ=255)`  
                                                                                                                                                                                                                                                    `agcgagcgCTGAGCGAAGATACTGCTTATGGGTAACACAGTGCTTTTGGCACCGAGGGTACACATCATTATCACCCGTGGTGACTTTGAGAAAGCAATAGACAAGAAACGTCCGGTTCTTAGTGTGGAAGTGACCCGCTTCCCGTTCGTCCGTCTGTTACTCAAACGAATCAAGGAGGTGTTCTGAGGGGACTGTTAGATGGTGAAGCCTGGGAAAAAGAAAACCCGCCAGTACAAGCAACTGGGTGTATAGCTTGCTTAGAGAAAGATGACCGTTATCCACACACCTGTAACAAAGGAGc                                                          <  1:50715/301‑1 (MQ=255)`  
                                                                                                                                                                                                                                                    `agcgagcgCCGAGCTAAGATACCTCTTATTGGTAACACAGTTCCTTTGGCACCGAGCGTCCACATCATTATCACCCGTGGTTACTTTGAGAAAGCAATAGACAAGAAACGTCCGGTTCTTAGTGTGGCAGTGACCCGCTTCCCGTTCGTCCGTCTGTTACTCAAACGAATCAAGGAGGTGTTCTGATGGGACTGTTAGATGGTGAAGCCTGGGAAAAAGAAAACCCGCCAGTACAAGCAACTGGGTGTATAGCTTGCTTAGAGAAAGATGACCGTTATCCACACACCTGTAACAAAGGAGc                                                          >  1:315748/1‑301 (MQ=255)`  
                                                                                                                                                                                                                                                    `agcgagcgCCGAGCTAAGATACCTCTTATTGGTAACACAGTTCCTTTGGCACCGAGCGTCCACATCATTATCACCCGTGGTGACTTTGAGAAAGCAATAGACAAGAAACGTCCGGTTCTTAGTGTGGCAGTGACCCGCTTCCCGTTCGTCCGTCTGTTACTCAAACGAATCAAGGAGGTGTTCTGATGGGACTGTTAGATGGTGAAGCCTGGGAAAAAGAAAACCCGCCAGTACAAGCAACTGGGTGTATAGCTTGCTTAGAGAAAGATGACCGTTATCCACACAccctgtctcttataca                                                          >  1:302802/1‑287 (MQ=255)`  
                                                                                                                                                                                                                                                                `gCTAAGATACCTCTTATTGGTAACACAGTTCCTTTGGCACCGAGCGTCCACATCATTATCACCCGTGGTGACTTTGAGAAATCAATATACAAGAAACGTCCGGTTCTTAGTGTGGCAGTGACCCGCTTCCCGTTCGTCCGTCTGTTACTCAAACGAATCAAGGAGGTGTTCTGATGGGACTGTTAGATGGTGAAGCCTGGGAAAAAGAAAACCCGCCAGTACAAGCAACTGGGTGTATAGCTTGCTTAGAGAAAGATGACCGTTATCCACACACCTGTAACAAAGGAGCTAACGATATGAc                                              >  1:224072/1‑301 (MQ=255)`  
                                                                                                                                                                                                                                                                   `aaGATACCTCTTATTGGTAACACAGTTCCTTTGGCACCGAGCGTCCACATCATTATCACCCGTGGTGACTTTGAGAAAGCAATAGACAAGAAACGTCCGGTTCTTAGTGTGGCAGTGACCCGCTTCCCGTTCGTCCGTCTGTTACTCAAACGAATCAAGGAGGTGTTCTGATGGGACTGTTAGATGGTGAAGCCTGGGAAAACGAAAACCCGCCAGTACAAGCAACTGGTTGTATAGCTTGCTTAGAGAAAGATGCCCGTTATCCCCACACCTTTAACAAAGTAGCTAACGATATGACCGa                                           >  1:319756/1‑301 (MQ=255)`  
                                                                                                                                                                                                                                                                    `aGATACCTCTTATTGGTAACACAGTTCCTTTGGCTCCGAGCGTCCACATCATTATCACCCGTGGTGACTTTGAGAAAGCAATAGACAAGAAACGTCCGGTTCTTAGTGTGGCAGTGACCCGCTTCCCGTTCGTCCGTCTGTTACTCAAACGAATCAAGGAGGTGTTCTGATGGGACTGTTAGATGGTGAAGCCTGGGAAAAAGAAAACCCGCCCGTACAAGCAACTGGGTGTATAGCTTGCTTAGAGAAAGATGACCGTTATCCACACACCTGTAACACAGGAGCTAACGATATGACCGaa                                          >  1:75759/1‑301 (MQ=255)`  
                                                                                                                                                                                                                                                                                  `ggTAACACAGTTCCTTTGGCACCGAGCGTCCACATCATTATCACCCGTGGTGACTTTGAGAAAGCAATAGACAAGAAACGTCCGGTTCTTAGTGTGGCAGTGACCCGCTTCCCGTTCGTCCGTCTGTTACTCAAACGAATCAAGGAGGTGTTCTGATGGGACTGTTAGATGGTGAAGCCTGGGAAAAAGAAAACCCGCCAGTACAAGCAACTGGGTGTATAGCTTGCTTAGAGAAAGATGACCGTTATCCACACACCTGTAACAAAGGAGCTAACGATATGACCGAACGTGAACAAGAgat                            >  1:100221/1‑301 (MQ=255)`  
                                                                                                                                                                                                                                                                                           `gTTCCTTTGGCACCGAGCGTCCACATCATTATCACCCGTGGTGACTTTGAGAAAGCAATAGACAAGAAACGTCCGGTTCTTAGTGTGGCAGTGACCCGCTTCCCGTTCGTCCGTCTGTTACTCAAACGAATCAAGGAGGTGTTCTGATGGGACTGTTAGATGGTGAAGCCTGGGAAAAAGAAAACCCGCCAGTACAAGCAACTGGGTGTATAGCTTGCTTAGAGAAAGATGACCGTTATCCACCCCCCTGTAACAAAGGAGCTAACGATATGACCGAACGTCAACACGAGATGATCATTaa                   >  1:56357/1‑301 (MQ=255)`  
                                                                                                                                                                                                                                                                                            `ttCCTTTGGCACCGGGCGTCCACATCATTATCACCCGTGGTGACTTTGAGAAAGCAATAGCCAAGAAACGTCCGGTTCTTAGTGTGGCAGTGACCCGCTTCCCGTTCGTCCGTCTGTTACTCAAACGAATCAAGGAGGTGTTCTGATGGGACTGTTAGATGGTGAAGCCTGGGAAAAAGAAAACCCGCCAGTACAAGCAACTGGGTGTATAGCTTGCTTAGAGAAAGATGACCGTTATCCACACACCTGTAACAAAGGAGCTAACGATATGACCGAACGTGAACAAGAGATGATCATTAAg                  <  1:132721/301‑1 (MQ=255)`  
                                                                                                                                                                                                                                                                                             `tCCTTTGGCACCGAGCGTCCACATCATTATCACCCGTGGTGACTTTGAGAAAGCAATAGACAAGAAACGTCCGGTTCTTAGTGTGGCAGTGACCCGCTTCCCGTTCGTCCGTCTGTTACTCAAACGAATCAAGGAGGTGTTCTGATGGGACTGTTAGATGGTGAAGCCTGGGAAAAAGAAAACCCGCCAGTACAAGCAACTGGGTGTATAGCTTGCTTAGAGAAAGATGACCGTTATCCACACACCTGTAACAAAGGAGCTAACGATATGACCGAACGTGAACAAGAGATGATCATTAAGt                 <  1:229456/301‑1 (MQ=255)`  
                                                                                                                                                                                                                                                                                             `tCCTTTGGCACCGAGCGTCCACATCATTATCACCCGTGGTGACTTTGAGAAAGCAATAGACAAGAAACGTCCGGTTCTTAGTGTGGCAGTGACCCGCTTCCCGTTCGTCAGTCTGTTACTCAAACGAATCAAGGAGGTGTTCTGATGGGACTGTTAGATGGTGAAGCCTGGGAAAAAGAAAACCCGCCAGTACAAGCAACTGGGTGTATAGCTTGCTTAGAGAAAGATGACCGTTATCCACACACCTGTAACAAAGGAGCTAACGATATGACCGAACGTGAACAagctgtcacttatacaa                 >  1:8959/1‑286 (MQ=255)`  
                                                                                                                                                                                                                                                                                             `tCCTTTGGCACCGAGCGTCCACATCATTATCACCCGTGGTGACTTTGAGAAAGCAATAGACAAGAAACGTCCGGTTCTTAGTGTGGCAGTGACCCGCTTCCCGTTCGTCAGTCTGTTACTCAAACGAATCAAGGAGGTGTTCTGATGGGACTGTTAGATGGTGAAGCCTGGGAAAAAGAAAACCCGCCAGTACAAGCAACTGGGTGTATAGCTTGCTTAGAGAAAGATGACCGTTATCCACACACCTGTAACAAAGGAGCTAACGATATGACCGAACGTGAACAAGAGATGATCATTAAGt                 <  1:287558/301‑1 (MQ=255)`  
                                                                                                                                                                                                                                                                                             `tCCTTTGGCACCGAGCGTCAACATCATTATCACCCGTGGTGACTTTGAGAAAGCAATAGACAAGAAACGTCCGGTTCTTAGTGTGGCAGTGACCCGCTTCCCGTTCGTCCGTCTGTTACTCAAACGAATCAAGGAGGTGTTCTGATGGGACTGTTAGATGGTGAAGCCTGGGAAAAAGAAAACCCGCCAGTACAAGCAACTGGGTGTATAGCTTGCTTAGAGAAAGATGACCGTTATCCACACACCTGTAACAAAGGAGCTAACGATATGACCGAACGTGAACAAGAGATGATCATTAAGt                 <  1:78824/301‑1 (MQ=255)`  
                                                                                                                                                                                                                                                                                              `ccTTTGGCACCGAGCGTCCACATCATTATCACCCGTGGTGACTTTGAGAAAGCAATAGACAAGAAACGTCCGGTTCTTAGTGTGGCAGTGACCCGCTTCCCGTTCGTCCGTCTGTTACTCAAACGAATCAAGGAGGTGTTCTGATGGGACTGTTAGATGGTTAAGCCTGGGAAAAAGAAAACCCGCCAGTACAAGCAACTGGGTGTATAGCTTGCTTAGAGAAAGATGACCGTTATCCACACACCTGTAACAAAGGAGCTAACGATATGACCGAACGTGAACAAGAGATGATCATTAAGtt                >  1:232057/1‑301 (MQ=255)`  
                                                                                                                                                                                                                                                                                                     `cACCGAGCGTCCACATCATTATCACCCGTGGTGACTTTGAGAAAGCAATAGACAAGAAACGTCCGGTTCTTAGTGTGGCAGTGACCCGCTTCCCGTTCGTCCGTCTGTTACTCAAACGAATCAAGGAGGTGTTCTGATGGGACTGTTAGATGGTGAAGCCTGGGAAAAAGAAAACCCGCCAGTACAAGCAACTGGGTGTATAGCTTGCTTAGAGAAAGATGACCGTTATCCACACACCTGTAACAAAGGAGCTAACGATATGACCGAACGTGAACAAGAGATGATCATTAAGTTGATAGAc         <  1:268595/301‑1 (MQ=255)`  
                                                                                                                                                                                                                                                                                                         `gAGCGTCCACATCATTATCACCCGTGGTGACTTTGAGAAAGCAATAGACAAGAAACGTCCGGTTCTTAGTGTGGCAGTGACCCGCTTCCCGTTCGTCCGTCTGTTACTCAAACGAATCAAGGAGGTGTTCTGATGGGACTGTTAGATGGTGAAGCCTGGGAAAAAGAAAACCCGCCAGTACAAGCAACTGGGTGTATAGCTTGCTTAGAGAAAGATGACCGTTATCCACACACCTGTAACAAAGGAGCTAACGATATGACCGAACGTGAAAAAGAGCTGATCATTAAGTGGATAGACaata     >  1:226334/1‑301 (MQ=255)`  

                                                                                                                                                                                                                                                                                                            `|`                                                                                                                                                                                                                                                                                                              
`TGCGTGTGGAGTATAGTTAACTGGTAATACGACTCACTAAAGGAGGTACACACCATGATGTACTTAATGCCATTACTCATCGTCATTGTAGGATGCCTTGCGCTCCACTGTAGCGATGATGATATGCCAGATGGTCACGCTTAATACGACTCACTAAAGGAGACACTATATGTTTCGACTTCATTACAACAAAAGCGTTAAGAATTTCACGGTTCGCCGTGCTGACCGTTCAATCGTATGTGCGAGCGAGCGCCGAGCTAAGATACCTCTTATTGGTAACACAGTTCCTTTGGCACCGAGCGTCCACATCATTATCACCCGTGGTGACTTTGAGAAAGCAATAGACAAGAAACGTCCGGTTCTTAGTGTGGCAGTGACCCGCTTCCCGTTCGTCCGTCTGTTACTCAAACGAATCAAGGAGGTGTTCTGATGGGACTGTTAGATGGTGAAGCCTGGGAAAAAGAAAACCCGCCAGTACAAGCAACTGGGTGTATAGCTTGCTTAGAGAAAGATGACCGTTATCCACACACCTGTAACAAAGGAGCTAACGATATGACCGAACGTGAACAAGAGATGATCATTAAGTTGATAGACAATAATG  >  NC_001604.1/7737‑8337`

  

Alignment Legend

Aligned base mismatch/match (shaded by quality score): `ATCG/ATCG` `< 3 ≤` `ATCG/ATCG` `< 10 ≤` `ATCG/ATCG` `< 22 ≤` `ATCG/ATCG` `< 34 ≤` `ATCG/ATCG` `< 38 ≤` `ATCG/ATCG`

Unaligned base: `atcg`    Masked matching base: `atcg`    Alignment gap: `‑`    Deleted base: `‑`

Reads not counted as support for junction

read\_name Not counted due to insufficient overlap past the breakpoint.

read\_name Not counted due to not crossing MOB target site duplication.
